# Draft Genome Sequence of *Mycobacterium bovis* 04-303, a Highly Virulent Strain from Argentina

**DOI:** 10.1128/genomeA.00931-13

**Published:** 2013-11-27

**Authors:** Christiane Nishibe, Ana Beatriz Canevari Castelão, Ricardo Dalla Costa, Beatriz Jeronimo Pinto, Leonardo Varuzza, Angel Adrian Cataldi, Amelia Bernardelli, Fabiana Bigi, Federico Carlos Blanco, Martín José Zumárraga, Nalvo Franco Almeida, Flábio Ribeiro Araújo

**Affiliations:** School of Computing, UFMS, Campo Grande, Mato Grosso do Sul, Brazila; Programa de Pós-Graduação em Ciência Animal, UFMS, Campo Grande, Mato Grosso do Sul, Brazilb; Life Technologies do Brasil, São Paulo, São Paulo, Brazilc; Instituto de Biotecnología, CICVyA, INTA-Castelar, Buenos Aires, Argentinad; Ceva Salud Animal, Ciudad Autónoma de Buenos Aires, Argentinae; Embrapa Beef Cattle, Campo Grande, Mato Grosso do Sul, Brazilf

## Abstract

*Mycobacterium bovis* strain 04-303 was isolated from a wild boar living in a free-ranging field in Argentina. This work reports the draft genome sequence of this highly virulent strain and the genomic comparison of its major virulence-related genes with those of *M. bovis* strain AF2122/97 and *Mycobacterium tuberculosis* strain H37Rv.

## GENOME ANNOUNCEMENT

Bovine tuberculosis (BT) is a chronic infectious disease caused by *Mycobacterium bovis*, affecting cattle (1), other domesticated species, wild animals, and humans (2, 3), leading to a worldwide agricultural loss of $3 billion each year (4). BT is also a zoonosis, being a concern in pastoral settings, especially in developing countries where the animal-human interaction and HIV prevalence are high (5). In wildlife, BT poses serious difficulties for control and eradication and contributes to the maintenance of the infection and its periodic spillover to domesticated animals (6).

This work reports the draft genome sequence of *M. bovis* strain 04-303, isolated from a wild boar in a free-ranging field in La Pampa, Argentina, in 2004. In mice, this strain induced the sudden development of pneumonia, massive areas of necrosis, and death after 6 weeks of infection, with two more lung colony-forming units than mice infected with the AN5 control strain. This strain has SB0140 spoligotyping and a 232224263322 mycobacterial interspersed repetitive unit (MIRU) pattern (7, 8).

The genome sequence was obtained using both MiSeq (9) and Ion Torrent sequencing (10) technologies, producing 521,134 paired-end reads and 651,617 reads after filtering, respectively, totaling 118 Mbp. We performed a reference-assisted genome assembly of filtered data of strain 04-303 with *M. bovis* AF2122/97 strain (4) (accession no. NC_002945) using Bowtie (11). There are 169 contigs (the largest one being 329,090 bp), with an N_50_ of 85,558 bp, a G+C content of 63%, and an average coverage of 27.3×.

Annotation with the NCBI PGAAP (12) identified a total of 3,988 protein-coding and 49 RNA genes. Phylogenetic and comparative analyses of 04-303 and four other genomes (those of *M. bovis* AF2122/97, *M. bovis* BCG strain Mexico, *Mycobacterium tuberculosis* H37Rv, and *Mycobacterium africanum* GM041182) showed that AF2122/97 is the closest strain, sharing 99.4% of its protein genes.

Comparisons were done among 04-303, *M. bovis* AF2122/97, and *M. tuberculosis* H37Rv (13). The orthologs of H37Rv *rv1759c* in 04-303 and AF2122/97 are pseudogenes. This gene encodes a PE-polymorphic GC-rich sequence (PGRS) protein that binds fibronectin, and alterations in this family might influence host or tissue tropism. The *rpfA* gene, associated with the resuscitation of dormant or nongrowing bacilli, showed an in-frame deletion of 240 bp in 04-303 and AF2122/97, leading to the synthesis of a shorter protein than that of H37Rv. The *pks6* gene, involved with synthesis of polyketines, is disrupted in 04-303 and AF2122/97. The inactivation of this gene has been shown to attenuate *M. tuberculosis* in a mouse model (14). The mammalian cell-entry *mce3* 6-gene operon is present in H37Rv and is absent in both 04-303 and AF2122/97. Curiously, *M. tuberculosis* strains disrupted in their *mce3* operon are attenuated in mice (15). The *esxA* (*esat6*) gene, which encodes a major virulence factor of *M. tuberculosis*, is conserved in AF2122/97, but a single mutation (A to G) was detected in 04-303, resulting in a threonine-to-alanine substitution (16).

### Nucleotide sequence accession numbers.

This whole-genome shotgun project has been deposited at DDBJ/EMBL/GenBank under the accession no. AVSW00000000. The version described in this paper is version AVSW01000000.
